# Changes in Nephritogenic Serum Galactose-Deficient IgA1 in IgA Nephropathy following Tonsillectomy and Steroid Therapy

**DOI:** 10.1371/journal.pone.0089707

**Published:** 2014-02-21

**Authors:** Junichiro Nakata, Yusuke Suzuki, Hitoshi Suzuki, Daisuke Sato, Tatsuya Kano, Hiroyuki Yanagawa, Keiichi Matsuzaki, Satoshi Horikoshi, Jan Novak, Yasuhiko Tomino

**Affiliations:** 1 Division of Nephrology, Department of Internal Medicine, Juntendo University Faculty of Medicine, Tokyo, Japan; 2 Department of Microbiology, University of Alabama at Birmingham, Birmingham, Alabama, United States of America; 3 Kyoto University Health Service, Kyoto, Japan; Institut national de la santé et de la recherche médicale (INSERM), France

## Abstract

**Background:**

Recent studies have shown that galactose-deficient IgA1 (GdIgA1) has an important role in the pathogenesis of IgA nephropathy (IgAN). Although emerging data suggest that serum GdIgA1 can be a useful non-invasive IgAN biomarker, the localization of nephritogenic GdIgA1-producing B cells remains unclear. Recent clinical and experimental studies indicate that immune activation tonsillar toll-like receptor (TLR) 9 may be involved in the pathogenesis of IgAN. Here we assessed the possibility of GdIgA1 production in the palatine tonsils in IgAN patients.

**Methods:**

We assessed changes in serum GdIgA1 levels in IgAN patients with clinical remission of hematuria and proteinuria following combined tonsillectomy and steroid pulse therapy. Further, the association between clinical outcome and tonsillar TLR9 expression was evaluated.

**Results:**

Patients (n = 37) were divided into two groups according to therapy response. In one group, serum GdIgA1 levels decreased after tonsillectomy (59%) alone, whereas in the other group most levels only decreased after the addition of steroid pulse therapy to tonsillectomy (41%). The former group showed significantly higher tonsillar TLR9 expression and better improvement in hematuria immediately after tonsillectomy than the latter group.

**Conclusions:**

The present study indicates that the palatine tonsils are probably a major sites of GdIgA1-producing cells. However, in some patients these cells may propagate to other lymphoid organs, which may partially explain the different responses observed to tonsillectomy alone. These findings help to clarify some of the clinical observations in the management of IgAN, and may highlight future directions for research.

## Introduction

Since the pathogenesis of immunoglobulin A nephropathy (IgAN) remains unclear [1], there is no specific therapy for this disease. Clinical evidence, particularly from kidney transplantation, indicates that the pathogenesis of IgAN is associated with an abnormality of the IgA immune system, rather than an abnormality of renal intrinsic cells [2] [3] [4] [5]. Episodic macroscopic hematuria, coinciding with mucosal infections of the upper respiratory tract [6] or an abnormal response to mucosal vaccination [7] [8] in IgAN patients indicates that dysregulation of the mucosal immune system is important in the pathogenesis of IgAN [9]. However, bone marrow (BM) or BM transplantation (BMT) studies in IgAN patients [10] [11] [12] [13] suggest that mucosal-type polymeric IgA is overproduced in systemic immune sites such as BM. In the 1980s, van Es et al. hypothesized that a “mucosa–BM axis” exists in which there is continuous trafficking of causative cells in the IgA immune system between mucosal sites and the BM [14] [15]. Clinical and experimental studies in the last decade have uncovered a detailed mechanism by which lymphocytes including mucosal B cells, travel between the mucosa and BM or lymphoid tissues [16] [17]. Although these findings support the mucosa-BM axis hypothesis, the causative cell and their origins remain unknown [18], precluding the development of disease-specific therapy for IgAN.

Our recent studies [19] [20] [21] have demonstrated that toll-like receptor (TLR), a key molecule in the innate and mucosal immunity, have pathological roles in IgAN in human and mice models. In particular, TLR9 activation appears to be important for the progression and severity of IgAN. Therefore, it is possible that mucosal cells expressing TLR9, such as tonsillar B cells [20] [21], may be involved in the pathogenesis. In addition, TLR activation also induces IgA switching in mucosal B cells [22]. These findings may explain why tonsillectomy has a favorable effect on the long-term renal survival in some IgAN patients [23] [24]. Other Japanese groups have also recently reported that tonsillectomy followed by steroid pulse therapy is more effective than tonsillectomy alone [25] [26] [27] [28]. It is speculated that steroid therapy may attenuate both inflammation and the number of causative lymphocytes that are capable of migrating into systemic site beyond the tonsil [9] [29]. However, none of these reports has provided data of any specific steroid therapy targets in IgAN.

Mesangial IgA present in the renal deposits is a subclass of IgA1 [30] and displays abnormal *O*-glycosylation [31] [32]. Furthermore, IgA1 molecules produced by tonsillar lymphocytes undergo aberrant *O*-glycosylation in IgAN patients [33] [34] [35]. Moldoveanu et al. [36] demonstrated that galactose-deficient IgA1 (GdIgA1) increases in the sera of IgAN patients using a *Helix aspersa* agglutinin (HAA) lectin assay, whereas recent studies have reported that GdIgA1 is nephritogenic in this disease [37] [38] [39] [40] [41]. We recently reported that serum GdIgA1 levels were clearly associated with disease activity in IgAN [42], indicating that serum GdIgA1 evaluation can be a potential non-invasive diagnostic and activity marker for IgAN [41] [42]. However, further evidence on the extent of GdIgA1-producing cells is necessary.

Our recent experimental findings indicate a role for cluster of differentiation 19-positive (CD19^+^) B cells: they appear to regulate nephritogenic IgA production, independent of T cells; they are disseminated in multiple lymphoid organs including BM and the spleen; and they are involved in IgAN [43].

The present study aimed to evaluate the changes in serum GdIgA1 levels in IgAN patients with complete remission of urinary abnormalities after tonsillectomy and steroid pulse therapy. We hypothesized that GdIgA1 is produced by the tonsils and should reduce after tonsillectomy.

## Subjects and Methods

We designed a cohort study to provide biological evidence of the patient types that show a decrease of serum GdIgA1 after tonsillectomy or additional steroid pulse therapy. The study protocol was approved by the Ethics Review Committee of Juntendo University Faculty of Medicine. Written informed consent was obtained from all patients.

### Patients

The inclusion criteria for our cohort were patients with IgAN diagnosed by renal biopsy who received both tonsillectomy and steroid pulse therapy. Patients were required to have clinical remission of hematuria [<5 red blood cells (RBC) per high power field (HPF)] and proteinuria (<0.15 g/g·Cr) after the 3 steroid pulse therapy sessions.

Serum GdIgA1 levels and clinical outcomes were analyzed before and after tonsillectomy, and after the first steroid pulse therapy session. The patients were divided into two groups according to their response to therapy: group A exhibited reduced serum GdIgA1 levels after tonsillectomy alone; group B showed no response to tonsillectomy alone. The patients in group B were further subdivided into those exhibiting reduced GdIgA1 levels after initiation of steroid therapy (group B1) and those not showing a reduction (group B2). The cut-off level for a reduction in GdIgA1 levels was 1 (after/before tonsillectomy or after the first steroid pulse therapy session/after tonsillectomy, <1 indicated a reduction; ≥1 indicated no reduction).

### Treatment protocol during the study period

Steroid pulse therapy (500 mg methylprednisolone intravenously [IV] per day for 3 consecutive days, thrice every 2 months) was administered from at least 2 weeks after the tonsillectomy. Between sessions of steroid pulse therapy, patients were prescribed oral prednisolone (0.5 mg/kg body weight) on alternate days.

### Analysis of serum and urine

Blood samples were obtained from patients just before tonsillectomy and before the first and second sessions of steroid pulse therapy. Serum GdIgA1 levels were measured by enzyme-linked immunosorbent assay. Costar 96-well U-bottom plates (Corning Inc., Corning, NY, USA) were coated at 4°C overnight with a F(ab')2 fragment of goat anti-human IgA (Jackson ImmunoResearch Laboratories, Inc., West Grove, PA, USA) at a 3 μg/ml. Plates were blocked at 4°C overnight with 2% bovine serum albumin (Sigma-Aldrich, St Louis, MO, USA) in phosphate-buffered saline containing 0.05% Tween 20 (v/v). Samples diluted in blocking buffer were added to each well and incubated at 4°C overnight. The captured IgA was subsequently desialylated by treatment at 37°C for 3 h with 10 mU/ml neuraminidase from *Vibrio cholera* (Roche Diagnostics Corp., Indianapolis, IN, USA) in 10 mM sodium acetate buffer, pH  = 5 [44]. Samples with and without neuraminidase treatment were analyzed in parallel to assess the proportion of molecules with sialylated and terminal *N*-acetylgalactosamine (GalNAc) residues. Samples were incubated at 37°C for 3 h with GalNAc-specific biotinylated HAA lectin (Sigma-Aldrich) diluted 1:500 in a blocking buffer [36] [45]. The bound lectin was detected by an avidin-horseradish peroxidase conjugate (ExtrAvidin; Sigma-Aldrich), and the reaction was developed with the peroxidase chromogenic substrate *o*-phenylenediamine-H_2_O_2_ (Sigma-Aldrich). The color reaction was stopped with 1 M sulfuric acid and absorbance at 490 nm was measured using an EL312 BioKinetics microplate reader (BioTek Instruments Inc., Winooski, VT, USA). HAA reactivity of IgA1 was then calculated in each sample as optical density (OD) units per 1 μg of IgA1. Naturally purified GdIgA1 from the plasma of an IgA1 multiple myeloma patient [36] [46] was treated with neuraminidase and used as the standard. The OD units per 1 μg neuraminidase-treated IgA1 were assigned a value of 100%. Total GdIgA1, expressed in U/ml of serum, was calculated as follows: (%× μg IgA/ml)/100 [47].

Urea nitrogen, creatine (Cr) and IgA in sera were measured. Urine samples were obtained at the same time, and urinary protein and RBC levels were assessed. We used the median RBC count per HPF for hematuria classification: 1–5/HPF (3/HPF), 6–10/HPF (8/HPF), 11–15/HPF (13/HPF), 16–20/HPF (18/HPF), 21–25/HPF (23/HPF), 26–30/HPF (28/HPF), and >30/HPF (33/HPF).

### Analysis of tonsils

TLR9 transcriptional levels in tonsils were measured by real-time reverse transcription-polymerase chain reaction (RT-PCR). Total RNA was extracted using the RNeasy Mini Kit (Qiagen GmbH, Hilden, Germany) according to the manufacturer's instructions. Real-time RT-PCR was performed using the 7500 Real-Time RT-PCR System with TaqMan Gene Expression Master Mix and TaqMan Gene Expression Assay (TLR9; Hs00370913_s1, Applied Biosystems, Foster City, CA, USA). The samples were compared with the GAPDH PCR product for quantification (Hs02786624_g1).

### Statistical analysis

Associations between different parameters were analyzed by the *t*-test. Analysis of variance was used to determine differences in the characteristics among the multiple groups. Data are expressed as mean ± SD or median values. P<0.05 was considered significant. All statistical analyses were performed using the Windows version of StatView 5.0 software (Abacus Concepts Inc., Berkeley, CA, USA).

## Results

**Figure 1:**
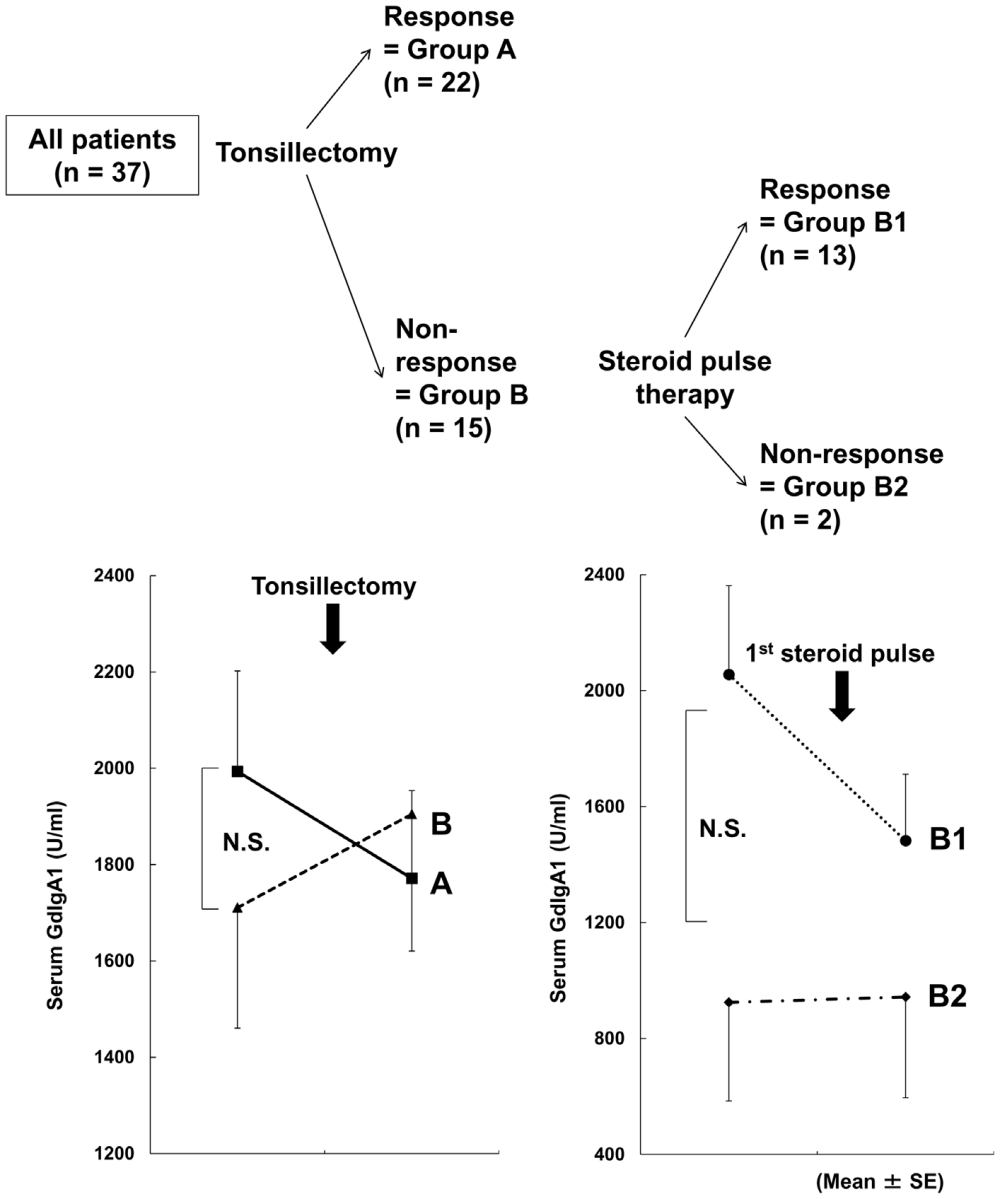
Classification of patients with immunoglobulin A nephropathy (IgAN) based on therapy response. Group A exhibited reduced serum galactose-deficient IgA1 (GdIgA1) levels after tonsillectomy alone. Group B showed no response to tonsillectomy alone. Group B patients were further subdivided into those exhibiting reduced GdIgA1 levels after initiation of steroid therapy (group B1) and those not showing a reduction (group B2). The cut-off level for a reduction in GdIgA1 levels was 1 (after/before tonsillectomy or after the first steroid pulse therapy session/after tonsillectomy, <1 indicated a reduction; ≥1 indicated no reduction).

We include 37 patients (16 males) according to the inclusion criteria, and their clinical profiles immediately before tonsillectomy are summarized in Table 1. Group A (responders tonsillectomy alone) comprised 22 patients, whereas group B (non-responders tonsillectomy alone) comprised 15 patients. In group B, GdIgA1 reduced after initiation of steroid therapy in 13 patients (group B1), and remained elevated in 2 patients (group B2; Figure 1). Although group B showed significantly lower serum Cr levels than group A, they were within the normal range. In addition, there were no significant differences in urinary proteinuria between the two groups (Table 1).

**Table 1:** Profiles of patients with immunoglobulin A nephropathy (IgAN) patients just before tonsillectomy.

	total (n = 37)	A (n = 22)	B (n = 15)	B1 (n = 13)	B2 (n = 2)
Duration from onset to tonsillectomy (year)*	5 (2–10)	5.5 (2–8)	4 (2–12)	7 (2–12)	2.5 (2.25–2.75)
Age at tonsillectomy (years old)	31.43±7.72	31.55±8.20	31.27±8.20	31.69±8.36	28.50±9.19
Male (%)	43 (16: 21)	45 (10: 12)	40 (6: 9)	38 (5: 8)	50 (1: 1)
Serum Cr (mg/dl)	0.83±0.24	0.91±0.26	0.69±0.16†	0.69±0.18	0.69±0.11
BUN (mg/dl)	12.86±3.41	13.55±3.45	11.79±2.49	12.08±2.57	10.00±0.00
Serum IgA (mg/dl)	323.3±99.1	326.8±88.2	317.9±112.8	325.3±120.5	273.5±29.0
Serum GdIgA1 (U/ml)	1878.8±971.2	1993.4±1024.7	1710.6±968.9	1834.5±977.6	878.8±278.9
Proteinuria / urine Cr (g/g·Cr)*	0.58 (0.29–0.85)	0.51 (0.2–0.68)	0.82 (0.51–1.13)	0.83 (0.71–1.36)	0.4 (0.35–0.44)
Hematuria (/HPF)	26.38±10.21	28.68±9.62	23.00±11.34	23.00±11.55	23.00±14.14
1–5/HPF	1 (2.7%)		1 (6.7%)	1 (7.7%)	
6–10/HPF	5 (13.5%)	3 (13.6%)	2 (13.3%)	2 (15.4%)	
11–15/HPF	2 (5.4%)		2 (13.3%)	1 (7.7%)	1 (50.0%)
16–20/HPF	1 (2.7%)		1 (6.7%)	1 (7.7%)	
21–25/HPF	3 (8.1%)	2 (9.1%)	1 (6.7%)	1 (7.7%)	
26–30/HPF	1 (2.7%)		1 (6.7%)	1 (7.7%)	
>30/HPF	24 (64.9%)	17 (77.3%)	7 (46.6%)	6 (46.1%)	1 (50.0%)

Data presented as Mean ± SD or.

*Median (interquartile range).

† p<0.05 (compared with group A). BUN, blood urea nitrogen; Cr, creatinine; GdIgA1, galactose-deficient IgA1; HPF, high power field; IgA, immunoglobulin A.

**Figure 2:**
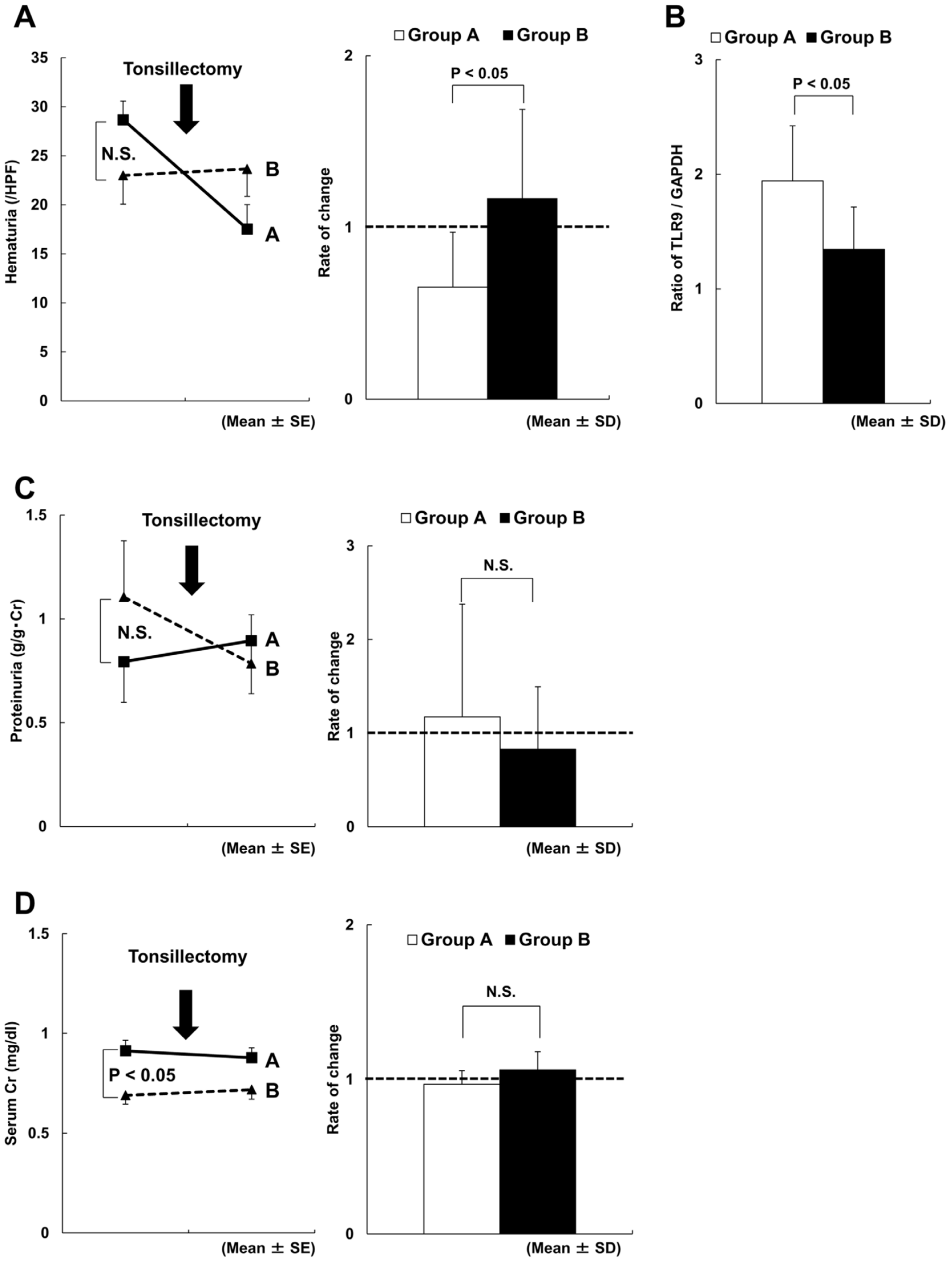
Comparison between groups A and B before and after tonsillectomy. (A) The rate of change in hematuria was <1 in group A and was significantly lower than that in group B. (B) Tonsillar TLR9 expression in group A was significantly higher than that in group B. (C) The rate of change in proteinuria before and after tonsillectomy was not significantly different between groups A and B. (D) The rate of change in serum Cr before and after tonsillectomy was not significantly different between groups A and B.

The ratio of changes in hematuria between immediately before and immediately after tonsillectomy in group A was significantly lower than that in group B (Figure 2A). As shown, the mean ratio after/before tonsillectomy in groups A and B were 0.63±0.34 and 1.23±0.66, respectively (P<0.05), suggesting improvement in hematuria after tonsillectomy alone, with a concurrent reduction in serum GdIgA1 levels in group A. Moreover, tonsillar TLR9 expression in group A was significantly higher than that in group B (Figure 2B), indicating that the responding patients comprised those with high tonsillar TLR9 expression. However, the rate of changes in proteinuria and serum Cr, before and after tonsillectomy, were not significantly different between groups A and B (Figures 2C and 2D).

**Figure 3:**
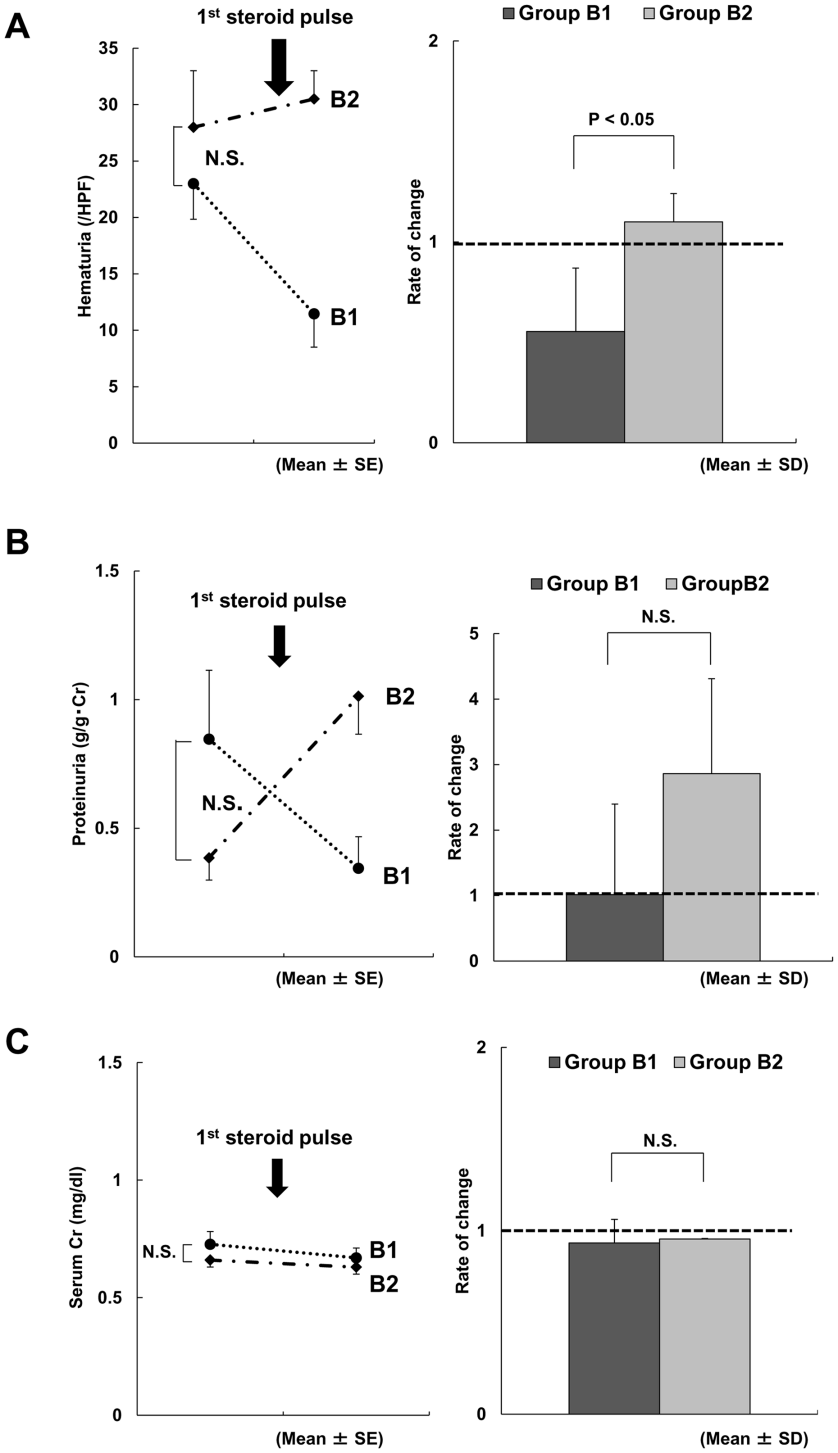
Comparison between groups B1 and B2 before and after the first steroid pulse therapy session. (A) An improvement in hematuria was observed in group B1, but not in group B2. The rate of change in hematuria in group B1 was significantly lower than that in group B2. (B) The rate of change in proteinuria before and after the first steroid pulse therapy session was not significantly different between groups B1 and B2. (C) The rate of change in serum creatinine before and after the first steroid pulse therapy session was not significantly different between groups B1 and B2.

After the first steroid pulse therapy session, group B1 showed improvements in both hematuria and serum GdIgA1 levels, despite the hematuria not improving after tonsillectomy alone (Figure 3A). In contrast, group B2 patients demonstrated no reduction in serum GdIgA1 levels after the first steroid pulse therapy session. No significant differences between groups B1 and B2 were observed in the rate of changes in proteinuria and serum Cr, before and after the first steroid pulse therapy session (Figures 3B and 3C).

## Discussion

Renal biopsy and immunohistochemical analysis of renal tissues remain the gold standard for diagnosing IgAN and evaluating acute lesions. However, new, safe, and reasonably specific non-invasive tests are emerging. Recent clinical studies suggest that elevated circulating GdIgA1 could offer a role in the diagnosis and evaluation of disease activity in IgAN [31] [32] [33] [34] [35] [36] [37] [38] [41] [42], because it appears to be involved in the pathogenesis of IgAN [48]. Other research suggests that β1,3-galactosyltransferase, the key enzyme for galactosylation of hinge-region IgA1 *O*-glycans, is downregulated in the tonsillar B cells of IgAN patients [49]. It is therefore possible that this alteration of enzyme activity contributes to the production of GdIgA1.

The present data demonstrate that serum GdIgA1 levels and hematuria decreased in 59% (22/37) of patients receiving tonsillectomy alone (group A), suggesting that the palatine tonsils may be a major site contributing to serum GdIgA1 levels. However, some patients (group B) showed improved hematuria and serum GdIgA1 levels following steroid pulse therapy, suggesting that GdIgA1-producing cells may also be localized outside the tonsils.

It has been discussed why mucosal type of polymeric IgA is produced in bone marrow [50]. However, emerging data has revealed that mucosal B cells, even tonsillar B cells, can migrate from inductive mucosal sites to systemic effector sites including BM through guiding adhesion molecules and chemokine/chemokine receptors [16] [17]. In addition, our recent study showed that IgA1 secreted by Epstein–Barr virus (EBV)-immortalized B cells from the peripheral blood of IgAN patients was mostly polymeric with galactose-deficient sialylated *O*-glycans [39]. These findings support the theory that GdIgA1-producing B cells may travel between the tonsils and systemic lymphoid organs and produce the nephritogenic IgA outside of mucosal sites; this possibly occurs through high endothelial venules (HEV), the lymphatics or the peripheral circulation [16] [17] [18].

The differences observed in the therapeutic responses of hematuria and serum GdIgA1 levels suggest that the number of GdIgA1-producing cells in lymphoid tissues vary among different tissues and among different IgAN patients. The extent of dissemination of GdIgA1-producing cells from mucosal to systemic sites may therefore vary among patients and may represent a determining factor for the efficacy of tonsillectomy with or without steroid pulse therapy.

Group A patients showed a decrease in GdIgA1 after tonsillectomy, but did not show a dramatic improvement in proteinuria. This may be due to short evaluation interval of 2–3 weeks between the tonsillectomy and the first steroid pulse therapy in our treatment protocol. In contrast, hematuria improved in group A despite the short duration. When we consider that the half-life of serum IgA is approximately 5 days, this finding suggests that renal injury and hematuria may relate to the glomerular deposition of GdIgA1 itself. In this regard, one can speculate that serum levels of GdIgA1 may influence renal pathology. Based on this principle, we undertook a preliminary examination of the correlation between these with present serum samples, but failed to find any significant correlation (data not shown). In this study, serum GdIgA1 levels were measured just before tonsillectomy. However, the intervals from renal biopsy to tonsillectomy are different for each individual. Further studies with serum samples collected at renal biopsy are required to assess this issue.

Clinical evidence suggests that acute tonsillitis and an upper respiratory tract infections are associated with IgAN exacerbation, suggesting that mucosal encounters with exogenous antigens may activate the specific tonsillar cells and contribute to disease progression. In this regard, TLR may be involved in the process [19] [20] [21] [51]; we have previously demonstrated that TLR9 expression, particularly in the mucosa, is associated with the progression of IgAN humans and mice [19] [20] [21]. Recently, we also reported that tonsillar TLR9 expression levels are associated with IgAN severity and response to tonsillectomy [20]. Patients with high TLR9 expression showed stronger and earlier remission of hematuria and proteinuria compared with those with a low TLR9 expression. In addition, patients whose serum IgA levels decreased more than average after tonsillectomy (large ΔIgA) showed higher cumulative proteinuria remission rates than patients with a smaller decrease in these levels (small ΔIgA). In this study, tonsillar TLR9 expression was significantly higher in the IgAN patients with reduced hematuria and serum GdIgA1 levels, compared with patients not responding to tonsillectomy alone.

TLR9 is expressed on B cells, and its activation is involved in IgA altering mucosal B cells through BAFF/APRIL (B-cell activating factor/a proliferation-inducing ligand) [22]. Thus, in combination with this study, this data may suggest that TLR9-positive B cells are involved in GdIgA1 production. Humans have two subclasses of IgA, namely IgA1 and IgA2. IgA1 contains *O*-glycosylation sites, whereas IgA2 and mouse IgA do not. Thus, the mouse model may exclude the aberrant *O*-glycosylation involved in human IgAN. Furthermore, recent studies suggest that aberrant *N*-glycan glycosylation may be involved in the pathogenesis of IgAN in mice [52] [53] [54]. We recently reported that aberrant glycosylation of IgA may be involved in the renal inflammatory cascade [55] and prognosis [56] of murine IgAN. These findings suggest that aberrant modifications of serum IgA carbohydrates are involved in the development of IgAN in both humans and mice, with *O*-glycans and *N*-glycans, respectively. Therefore, the pathogenesis of IgAN possibly overlaps in humans and mice in terms both of the genetic regulation [9] [56] [57] [58] and the underlying mechanisms, including the causative cell types and TLR9 expression [19] [21].

We recently reported that IgAN-prone mice with genetic and pathological features similar to human IgAN [9] [19] [21] [43] [55] [56] [57] [58] may have pathogenic B cells, but not matured plasma cells, and that they are disseminated to systemic lymphoid tissues such as BM and the spleen [43]. Furthermore, mucosal TLR9 activation appears to be involved in IgAN disease progression through nephritogenic IgA production in mice [21]. These experimental findings further suggest that mucosal/tonsillar TLR9 activation on B cells may be involved in the production of nephritogenic GdIgA1. However, before tonsillectomy the serum GdIgA1 levels were not significantly different between groups A and B, whereas the tonsillar TLR9 expression was significantly different. This finding may indicate that tonsillar TLR9 expression has a week influence on GdIgA1 production at non-tonsillar sites. In addition, because the severity of renal injury was not different between the two groups, the precise origin of GdIgA1 may be not important for its nephritogenicity. However, the mechanisms underlying the regulation of GdIgA1 production at non-tonsillar systemic sites should be evaluated in future studies.

This study was designed only as a preliminary study to test the hypothesis that that GdIgA1 is produced by the tonsils and should reduce after tonsillectomy. However, we can still draw several conclusions; we suggest that GdIgA1-producing cells may be localized to the palatine tonsils, and that the originating mucosal B cells then disseminate to systemic lymphoid organs. Although further research is necessary, these observations may account for the clinical observations that tonsillectomy is sufficient for some patients, whereas others require additional steroid therapy. To be effective in directing treatment, it is necessary to establish an objective method to evaluate the extent of systemic involvement. In the meantime, the current findings provide further clarification on the pathogenesis of IgAN.
